# Alcohol, Resistance Exercise, and mTOR Pathway Signaling: An Evidence-Based Narrative Review

**DOI:** 10.3390/biom13010002

**Published:** 2022-12-20

**Authors:** Danielle E. Levitt, Hui-Ying Luk, Jakob L. Vingren

**Affiliations:** 1 Department of Kinesiology & Sport Management, Texas Tech University, Lubbock, TX 79409, USA; 2 Department of Kinesiology, Health Promotion, & Recreation, University of North Texas, Denton, TX 76201, USA

**Keywords:** resistance exercise, alcohol, mTOR, protein synthesis, myopathy

## Abstract

Skeletal muscle mass is determined by the balance between muscle protein synthesis (MPS) and degradation. Several intracellular signaling pathways control this balance, including mammalian/mechanistic target of rapamycin (mTOR) complex 1 (C1). Activation of this pathway in skeletal muscle is controlled, in part, by nutrition (e.g., amino acids and alcohol) and exercise (e.g., resistance exercise (RE)). Acute and chronic alcohol use can result in myopathy, and evidence points to altered mTORC1 signaling as a contributing factor. Moreover, individuals who regularly perform RE or vigorous aerobic exercise are more likely to use alcohol frequently and in larger quantities. Therefore, alcohol may antagonize beneficial exercise-induced increases in mTORC1 pathway signaling. The purpose of this review is to synthesize up-to-date evidence regarding mTORC1 pathway signaling and the independent and combined effects of acute alcohol and RE on activation of the mTORC1 pathway. Overall, acute alcohol impairs and RE activates mTORC1 pathway signaling; however, effects vary by model, sex, feeding, training status, quantity, etc., such that anabolic stimuli may partially rescue the alcohol-mediated pathway inhibition. Likewise, the impact of alcohol on RE-induced mTORC1 pathway signaling appears dependent on several factors including nutrition and sex, although many questions remain unanswered. Accordingly, we identify gaps in the literature that remain to be elucidated to fully understand the independent and combined impacts of alcohol and RE on mTORC1 pathway signaling.

## 1. Introduction

Skeletal muscle (SKM) quantity is the result of two processes: muscle protein synthesis (MPS) and muscle protein breakdown (MPB). When the rate of MPS exceeds the rate of MPB, the net result is protein accretion (hypertrophy). A well-known, efficacious method of increasing muscle size is performance of repeated bouts of resistance exercise (RE). RE increases overall protein turnover by concomitantly increasing the rates of both MPS and MPB. Following a single bout of RE, the rate of MPS is increased above rest in the early recovery period (e.g., 1–5 h) [1,2,3] through at least 24 h [1,2] and up to 48 h [2] after exercise. Increases up to 2.7-fold have been reported [1,2,3]. Provided sufficient mechanical stimulation and nutrient intake, the increase in MPS exceeds that of MPB, resulting in net protein accretion [2,4,5], with MPS measured using primed constant infusions of [^2^H_5_]phenylalanine [2,4,5] and MPB measured using a primed constant infusion of 15N-phenylalanine [2] or phenylalanine rate of appearance in circulation [4,5]. This demonstrates that muscle hypertrophy associated with RE training is due to greater elevation in MPS than in MPB and not suppression of MPB.

Physically active individuals, especially those who regularly perform RE and vigorous-intensity exercise, report consuming alcohol more frequently and in larger quantities compared to sedentary individuals [6,7,8,9]. Unlike RE, alcohol (as ethanol) administration suppresses MPS. In rodent models, MPS in the gastrocnemius [10] and the plantaris [11] muscles was significantly reduced 2.5 h after ethanol administration. A significant reduction in MPS measured using ^35^S-methionine/cysteine incorporation, but no change in MPB measured using ^35^S-methionine/cysteine release, was also observed when human myocytes were cultured with ethanol or its major metabolites, acetaldehyde and acetate, for 24–72 h [12]. Furthermore, chronic at-risk alcohol use is associated with suppressed MPS compared to healthy controls [13], with no differences or possibly also a suppression in MPB measured by assessing the 3-methylhistidine/creatinine ratio in urine corrected for non-muscle-derived 3-methylhistidine and creatinine [14]. Together, these data suggest that myopathy (decreased muscle mass or function) associated with at-risk alcohol use is preferentially due to a reduction in MPS rather than an increase in MPB. These findings are highly relevant in physically active populations due to potentially impaired exercise adaptations. Moreover, muscle hypertrophy associated with RE training renders it an attractive, low-risk option for counteracting or preventing myopathy associated with alcohol use disorder. Elucidating the independent and combined effects of these stimuli on anabolic signaling in SKM will help inform the potential efficacy of exercise interventions to improve SKM health in people with at-risk alcohol use.

Protein synthesis is controlled, at least in part, by highly conserved cell signaling pathways. Key among these is the mechanistic/mammalian target of rapamycin (mTOR) complex 1 (mTORC1) signaling pathway. A variety of stimuli, including ethanol and RE, can modulate the activity of this pathway and thus contribute to increases or decreases in MPS. For example, administration of rapamycin, a potent inhibitor of mTORC1, to human subjects prevented the RE-induced increase in MPS observed in the early (1–2 h) post-exercise period, at least in part by blocking, attenuating, or delaying key events within the mTORC1 pathway [15]. Administration of rapamycin in rodents nearly completely (95%) blocked overload-induced hypertrophy [16], supporting the importance of mTORC1 pathway signaling for increasing muscle size with RE training.

Importantly, muscle hypertrophy results from repeated bouts of RE (i.e., training) that allow for a net protein accretion over time, thus the anabolic stimulus induced by each acute bout is important. Ethanol and RE can independently alter the mTORC1 pathway and MPS. When consumed before or after a bout of RE, ethanol can attenuate RE-induced mTORC1 pathway signaling and, therefore, ethanol ingestion might be detrimental to maximizing RE-induced MPS [17,18,19,20]. Because signaling through mTORC1 modulates key events in the translational process in general and is important for MPS specifically, the aims of this evidence-based review are to summarize up-to-date knowledge regarding (1) key events in the mTORC1 signaling pathway and their regulation, (2) modulation of mTORC1 signaling by acute ethanol, (3) modulation of mTORC1 signaling by RE, and (4) effects of alcohol on RE-induced mTORC1 signaling.

## 2. mTORC1 Pathway Signaling

### 2.1. Overview of the Pathway

The mTOR protein exists in two distinct multiprotein complexes. In complex 1 (mTORC1), mTOR is associated with the regulatory associated protein of mTOR (Raptor), proline-rich Akt substrate of 40-kDa (PRAS40), mammalian lethal with sec-13 (mLST8, also known as GβL), and DEP-domain-containing mTOR-interacting protein (Deptor). In complex 2 (mTORC2), mTOR is associated with the raptor-independent companion of mTOR (Rictor), mLST8, Deptor, protein observed with Rictor (Protor), and mammalian stress-activated protein kinase-interacting protein (mSin1). Each complex has unique upstream regulation and downstream targets. Rapamycin inhibits signaling through mTORC1 by binding to its intracellular receptor, FK506-binding protein 12 (FKBP12), and the subsequent FKBP12-rapamycin complex binds to the FKBP12-rapamycin binding (FRB) domain of mTOR [21]. This binding event limits interaction between mTOR and Raptor [22]. However, signaling through mTORC2 appears insensitive to inhibition by rapamycin [23,24,25] unless administered at very high concentrations (e.g., 1000 nM for inhibition of mTORC2 versus 1–10 nM for inhibition of mTORC1) [26,27,28] or for a prolonged time period (e.g., 24–72 h) [27,29]. Furthermore, signaling through mTORC1 is associated with cell growth and cell metabolic activity, whereas signaling through mTORC2 is associated with cytoskeletal maintenance and cell survival [25]. While less is known about its role, mTORC2 may contribute to muscle hypertrophy with RE training [30] in synergy with mTORC1 [31] and its activity appears decreased with ethanol [32]. However, this review will focus on mTORC1, and mTOR will hereafter refer to the protein within complex 1.

The mTOR signaling pathway is a nutrient, growth factor, and mechanically sensitive anabolic pathway that plays a key role in load-induced SKM hypertrophy. Different stimuli change the activation state of a variety of upstream components of the pathway to regulate the kinase activity of mTOR. Once activated, mTOR exerts its downstream effects. It should be noted that other pathways, such as the MAPK pathways, also promote protein synthesis and converge with the mTOR signaling pathway at several locations. Major outcomes of the activation of the mTOR signaling pathway include translation initiation, peptide elongation, ribosomal biogenesis, suppression of autophagy, and ultimately, protein synthesis (reviewed in [30,33]).

### 2.2. Control by Insulin and Growth Factors

In canonical mTOR pathway signaling, the ligands insulin and insulin-like growth factor-1 (IGF-1) bind and activate their membrane-bound receptors, resulting in signal transduction to phosphoinositide-3-kinase (PI3K) [34,35]. PI3K converts phosphatidylinositol (4,5) bisphosphate (PIP2) into phosphatidylinositol (3,4,5) trisphosphate (PIP3). PIP3 binds protein kinase B (Akt), and Akt translocates to the plasma membrane, where it is phosphorylated by 3-phosphoinositide-dependent-kinase 1 (PDK1) [36]. Once phosphorylated and activated, Akt is involved in a number of phosphorylation events important for mTOR activation including inhibition of tuberous sclerosis complex 2 (tuberin; TSC2) [37]. Inhibition of TSC2 appears to be a point of convergence for mTOR activation mediated by growth factors and mechanical stimuli.

TSC2 resides within a complex alongside tuberous sclerosis complex 1 (hamartin; TSC1) and TBC1D7 [38]. TSC2 is the only known GTPase activating protein (GAP) toward Ras homolog enriched in brain (Rheb); thus, when it is active, TSC2 promotes the hydrolysis of GTP to GDP by Rheb, maintaining Rheb in its inactive GDP-loaded state [39,40]. Akt activation results in inhibitory phosphorylation of TSC2 on 4 amino acid residues (Ser939, Ser1086, Ser1088, and Thr1422) [37], preventing interaction between TSC2 and Rheb, and allowing Rheb to remain in its active GTP-loaded state [39]. In this state, Rheb binds and activates mTOR at the lysosome [41,42,43]. This activation appears to be contingent on nutrient-stimulated activation of the lysosomally-anchored Rag GTPase heterodimer [44].

### 2.3. Control by Mechanical Stimuli

The specific upstream mechanism by which a mechanical stimulus (e.g., RE) transduces a signal to enable mTOR activation is poorly understood but attempts to elucidate this mechanism have provided substantial insight. The activation of mTOR in response to mechanical stimuli bypasses signaling through PI3K/Akt [45,46]. Instead, mechanically-driven mTOR pathway signaling occurs through a set of events that remains to be completely defined, but converges with the PI3K/Akt pathway at TSC2 [47,48]. Knockout of either TSC2 or Rheb attenuated mTOR signaling in response to lengthening (eccentric) muscle actions [47], indicating that TSC2 and Rheb are important but not solely responsible for load-induced mTOR pathway signaling. Recently, amino acid residues on TSC2 that are phosphorylated in response to lengthening contractions have been reported [47]. Such sites were independent of those known to be phosphorylated by Akt [47]. Although no single site was solely responsible for muscle load-induced changes in mTOR pathway signaling, analysis of the functional relevance of combined phosphorylation events could provide important insight into a mechanism underlying load-induced activation of mTOR pathway signaling [47]. Furthermore, mechanical stimuli induce spatial shift of TSC2 to allow mTOR to associate with GTP-loaded Rheb and the Rag complex [48,49]. Therefore, it appears that RE-induced signaling, as with canonical insulin or growth factor signaling, acts through removal of the GAP activity of TSC2. This allows for interaction between Rheb and mTOR to occur due to a change in location of the TSC2 protein such that it cannot interact with its target, Rheb, rather than inhibition of its intrinsic GAP activity.

The generation of phosphatidic acid (PA) also contributes to mechanically-induced stimulation of mTOR signaling [50]. Although the mechanisms are not fully understood, mTOR [51] and Deptor [52] each appear to recognize and bind PA, and PA appears involved in the lysosomal targeting of mTOR [53]. Initial observations suggested a necessary role for phospholipase D (PLD) in generating PA involved in eccentric contraction or stretch-induced mTOR activation [46,54]. However, PLD activity was unable to account for the sustained increase in PA concentrations after such mechanical stimulus [54,55]. Further investigation confirmed that PLD was not necessary for mTOR activation by PA in the context of mechanical stimulation; instead, diacylglycerol kinase (DGK)ζ appeared to be necessary for the mechanically-induced stimulation of mTOR by PA [56]. The efficacy of DGKζ in promoting PA-induced mTOR activation had been previously documented in HEK293 cells [57] and is necessary for SKM hypertrophy in response to mechanical overload [58]. Thus, although PLD might play some role in activating mTOR via generation of PA, DGKζ appears necessary to fully account for the generation of PA induced by eccentric muscle contraction. This concept should be further explored in response to more traditional and efficacious (e.g., concentric and eccentric) loading paradigms. For recent in-depth reviews on mechanotransduction, see references [59,60,61].

### 2.4. Control by Nutrients (Excluding Ethanol)

Nutrient availability affects signal transduction through the mTOR signaling pathway. Amino acids, particularly (but not solely) leucine, potently stimulate signaling through mTOR [62,63], although not through canonical PI3K/Akt [62] or TSC2 [64] signaling. Instead, Rag GTPases are required for amino acid-stimulated signaling through mTOR [44,65,66]. The Rag proteins exist as heterodimers (RagA or RagB and RagC or RagD) and are anchored to the lysosome by Ragulator [44], a scaffolding protein and guanine exchange factor toward Rag A/B [67]. Rag, in turn, binds Raptor, the companion of mTOR within complex 1 [66], in an amino acid-dependent manner. Dimers containing RagD rather than RagC preferentially recruit mTOR to the lysosome [68]. When components of Ragulator are mutated [44] or when Rag protein subunits have been knocked down [65], amino acids fail to stimulate S6K1^T389^ phosphorylation (i.e., by mTOR within complex 1). While the GTP-loading state of RagA/B mediates amino acid-dependent mTOR activation [44,65,66], heterodimers containing RagB are less dependent on amino acid stimulation than those containing RagA [68], but Rag B and D appear more critical for leucine-mediated mTOR signal transduction [69,70]. Under amino acid-poor conditions, the protein sub-complex GATOR1 exerts GAP activity toward RagA/B, stimulating GTP hydrolysis [71]. Under amino acid-rich conditions, GATOR2 (the counterpart of GATOR1) dissociates from Sestrin 1 [72], inhibits GATOR1 [69], and thus acts in a complementary manner to the Ragulator. Therefore, the Rag-Ragulator complex and GATOR2 activation are important for amino acid-stimulated mTOR pathway activation. For more in-depth reviews regarding the current knowledge of nutrient-dependent regulation of signaling through mTOR, see references [73,74,75].

Alternatively, signal transduction through mTOR is repressed under conditions of nutrient deficiency (e.g., high AMP:ATP ratio) or cellular stress (e.g., hypoxia). Under these cellular conditions, AMP-activated protein kinase (AMPK) is activated by phosphorylation on Thr172 [76] and, in turn, phosphorylates TSC2 and Raptor. When phosphorylated by AMPK on 3 amino acid residues (Thr1227, Ser1387, and Ser1345; activating events), TSC2 exerts GAP activity toward Rheb, removing Rheb-induced mTOR activation [77,78]. AMPK also phosphorylates the mTOR-associated protein Raptor on Ser722 and Ser729, inhibiting mTOR kinase activity [79]. Activation of AMPK and mTOR have largely opposite effects on MPS and MPB [80]; thus, it appears that the relative activation of the two proteins might be an important determinant of hypertrophic adaptations in SKM. For recent in-depth reviews regarding the nutrient sensing role of AMPK and its interaction with mTOR pathway signaling, see references [81,82,83].

### 2.5. Downstream of mTOR

Upon activation, mTOR phosphorylates its downstream substrates including ribosomal protein S6 kinase-1 (S6K1; Thr389) [84], an important intermediate in promoting translational efficiency, and eukaryotic translation initiation factor (eIF) 4E-binding protein-1 (4E-BP1; Thr37, Thr46, Ser65, and Thr70) [85,86,87], a translational repressor. The phosphorylation of one or both proteins directly downstream of mTOR (most commonly S6K1) is often used as a surrogate marker for mTOR activation state. Upon phosphorylation by mTOR, 4E-BP1 releases eIF4E [87], allowing eIF4E to form a heterotrimeric complex (eIF4F) with eIF4G and eIF4A. Within eIF4F, eIF4E recognizes and binds the 5′ cap structure on mRNA, eIF4A is an RNA helicase, and eIF4G is a scaffolding protein for the other components of the complex [88]. Thus, the phosphorylation and inhibition of 4E-BP1 by mTOR allows for 5′ cap-dependent translation initiation. Another initiation factor, eIF4B, promotes the helicase activity of eIF4A [89]. S6K1 is partially, but not solely, responsible for the phosphorylation and activation of eIF4B^Ser422^ [90], indicating a role for S6K1 activity in translation initiation. It should be noted that the phosphorylation of eIF4B^S422^ represents a point of convergence between the mTOR and MAPK signaling pathways [90] and that mTOR-independent 4E-BP1 phosphorylation has been reported [91]. Moreover, the time courses of the activation of these proteins may differ, so caution should be exercised when interpreting the meaning of surrogate markers.

Beyond its role in translation initiation, S6K1 also indirectly regulates peptide chain elongation and ribosomal biogenesis. S6K1 phosphorylates and inhibits eukaryotic elongation factor 2 kinase (eEF2K; Ser366) [92], which removes its inhibition of eukaryotic elongation factor 2 (eEF2; Thr56) [92,93], thus promoting peptide elongation. Specifically, eEF2 allows for the translocation of the growing peptide chain from the ribosomal A-site to the P-site by coupling the translocation with GTP hydrolysis [94]; a critical step in the process of protein synthesis. S6K1 also phosphorylates ribosomal protein S6 (rpS6; Ser235/236 and Ser240/244) [95,96,97], a protein within the small (40S) ribosomal subunit. In turn, rpS6 regulates cell size in an mTOR-dependent manner [98]. It should be noted that p90 ribosomal S6 kinases (RSKs) also phosphorylate eEF2K^S366^ [92] and rpS6^S235/236^ [96].

Overall, mTOR integrates signals from growth factors, nutrients, and mechanical stimuli. In stressful or nutrient-poor conditions, mTOR kinase is inactive. In nutrient-rich and anabolic environments, mTOR can phosphorylate its downstream substrates to promote translation. Two factors that can acutely alter the activation of various components of the mTOR signaling pathway are ethanol and RE. Current knowledge regarding the effects of each alone and the combined effects of ethanol and RE on mTOR pathway signaling will be discussed in subsequent sections.

## 3. Ethanol and Signaling through mTOR

**Figure 1:**
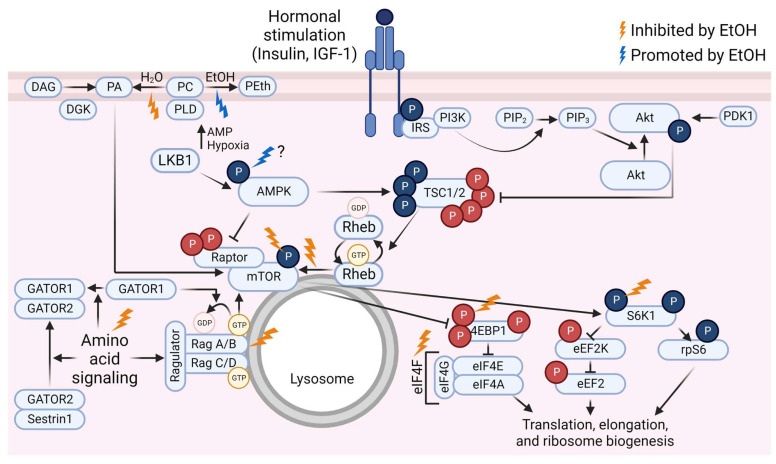
Overview of the mammalian/mechanistic target of rapamycin (mTOR) signaling pathway and events that are inhibited (gold lightning bolts) or promoted (blue lightning bolt) by ethanol (EtOH). Phosphate groups (P) in red indicate inhibitory events; P in blue indicate activating events. Abbreviations: 4E binding protein 1 (4EBP1), AMP-activated protein kinase (AMPK), diacylglycerol kinase (DGK), diacylglycerol (DAG), eukaryotic elongation factor (eEF), eukaryotic initiation factor (eIF), GAP activity toward Rags (GATOR), insulin receptor substrate (IRS), insulin-like growth factor-1 (IGF-1), liver kinase B1 (LKB1), p70 S6 kinase 1 (S6K1), phosphatidic acid (PA), phosphatidylcholine (PC), phosphatidylethanol (PEth), phosphatidylinositol (4,5)-bisphosphate (PIP2), phosphatidylinositol (3,4,5)-trisphosphate (PIP3), phosphoinositide 3-kinase (PI3K), phospholipase D (PLD), protein kinase B (Akt), pyruvate dehydrogenase kinase 1 (PDK1), Ras homolog enriched in brain (Rheb), ribosomal protein S6 (rpS6), tuberous sclerosis complex (TSC). Created using Biorender.com (accessed on 13 December 2022).

Although the exact mechanisms by which ethanol adversely affects MPS have not been fully established, a substantial amount of evidence exists to demonstrate that acute ethanol administration alters the activation state of a variety of key proteins within the mTOR signaling pathway. Furthermore, ethanol can alter the anabolic responses to stimuli such as leucine, insulin, and IGF-1 in such models. This portion of the review will focus on describing the ethanol-induced changes to mTOR pathway signaling in the basal state and in response to amino acids or hormone administration. Much of the literature relies on preclinical or cell culture-based models. Rodents have higher rates of ethanol clearance than humans [99,100], so dosages used in rodent studies do not necessarily translate directly to humans. However, the animals reach binge-level blood alcohol concentrations (BAC). In humans, consuming a quantity of ethanol to raise the BAC to at least 0.08 g/dL is considered a binge [101]. This equates to a minimum of roughly 4 or 5 standard drinks (14 g ethanol each) in 2 h for females or males, respectively [101], depending on body size and composition [102]. The dose of 100 mM ethanol frequently used in cell culture models is equivalent to a BAC of 0.46 g/dL which is typically supraphysiological [103], although similar or higher values have been reported [104,105,106]. Still, results from various rodent models of ethanol administration (reviewed in [107]) and cell culture-based models provide insight into potential mechanistic impacts of ethanol in human muscle. For a graphic summary of the ethanol-induced changes to mTOR pathway signaling, see Figure 1.

### 3.1. Ethanol Effects Upstream of mTOR

At least some ethanol-induced changes in mTOR pathway signaling can be attributed to inhibitory regulation upstream of mTOR. For example, C2C12 myocytes cultured with 100 mM ethanol (18–24 h) consistently display increased phosphorylation of AMPK^T172^ compared to control cells [77,108,109] with a concomitant increase in phosphorylation of TSC2^S1387^ [77,109]. Interestingly, co-incubation with leucine [77] or PA [109] abolishes the ethanol-induced increase in phosphorylation of AMPK^T172^ and TSC2^S1387^, indicating that it is possible for anabolic agents to “balance” the anti-anabolic effects of ethanol. It is important to note that ethanol can also interfere with the endogenous production of PA by preferentially converting phosphatidylcholine to phosphatidylethanol (reviewed in [110]), potentially impacting PA-mediated mechanotransduction. However, rodent studies using high-dose administration of ethanol (up to 5 g/kg) did not result in altered AMPK^T172^ phosphorylation [111,112,113] or binding of TSC1 with TSC2 [111,112] in the 2.5–4 h post-alcohol recovery period. With chronic ethanol feeding, the phosphorylation of LKB1 (directly upstream of AMPK) and AMPK are upregulated in the gastrocnemius in older but not younger adult rats [114]. Given the increased AMPK phosphorylation observed in myocytes cultured with ethanol and increased AMPK phosphorylation in SKM of aged rats chronically fed an ethanol-containing diet, it is possible that increased AMPK phosphorylation after acute ethanol administration might have been observed at time points other than those studied. It is also possible that the effects of ethanol on AMPK phosphorylation are readily apparent in cell culture due to the concentration and duration of ethanol exposure, whereas the same effects may simply take a greater number of exposures in vivo. Thus, further investigation of changes in intramuscular AMPK phosphorylation with acute ethanol administration is warranted.

AMPK can also suppress mTOR activity by phosphorylating the mTOR-associated protein, Raptor (Ser722 and Ser792), independently of TSC2 [79]. In myocytes incubated with ethanol that displayed increased phosphorylation of AMPK, there were concomitant increases in phosphorylation of Raptor^S792^ and mTOR/Raptor association [115]. Furthermore, ethanol administered to rats increased binding of mTOR and Raptor 2.5 h later, which was associated with a decrease in MPS regardless of age [112]. The relationship between mTOR/Raptor association and mTOR kinase activity is complex, as dissociation of Raptor from mTOR inhibits mTOR from phosphorylating a subset of its downstream substrates [22], whereas increased binding of mTOR and Raptor has also been observed under nutrient-poor conditions [116], indicating that the effect of mTOR/Raptor binding is most likely due to the strength and locations of the interaction between the two proteins [117]. It is possible that ethanol could induce enhanced binding of mTOR and Raptor such that recruitment of mTOR targets (S6K1 and 4E-BP1) is inhibited, thereby reducing mTOR signal transduction.

Ethanol also affects the ability of amino acids to stimulate signaling through mTOR [77,118]. This suppression appears to be mediated by an ethanol-induced decrease in binding of mTOR/Raptor with lysosomally-associated Rag proteins. Amino acids stimulate mTOR pathway signaling, at least in part, by promoting the appropriate GTP/GDP loading state of the Rag heterodimer and thus facilitating binding of Raptor/mTOR with the Rag complex, allowing for signal transduction. Ethanol prevented the feeding-induced increases in Rag A and C binding with mTOR, Sestrin1 binding with GATOR2, and the increase in MPS in SKM of male mice without altering amino acid availability or circulating insulin [119]. Co-culturing C2C12 myocytes with leucine and ethanol negated the effects of either substance alone on the association between RagA with mTOR such that the percent of mTOR associated with RagA was not different from that of control cells [77]. In contrast, the association of RagC with mTOR in the presence of leucine and ethanol was not statistically different from leucine alone; however, it was also not different from control cells. In the same investigation [77], culturing C2C12 myocytes expressing a constitutively active form of RagA/C with ethanol suppressed the phosphorylation of mTOR targets (S6K1 and 4E-BP1), indicating that ethanol likely prevents binding of mTOR (via Raptor) with the Rag proteins independently of GTP/GDP loading state. Although it appears plausible that the effects of ethanol on attenuation of leucine-induced stimulation of the mTOR pathway could be due, at least in part, to decreased binding between mTOR and the Rag complex, more work is needed to verify the effects of ethanol on Raptor/mTOR binding with Rag proteins in vivo and with more physiologically relevant doses of ethanol (e.g., 25–50 mM).

The Rag-Ragulator complex aids in mTOR signal transduction, at least in part, by tethering the mTOR/Raptor complex to the lysosome, where Rheb-GTP can bind and activate mTOR. Incubating C2C12 myocytes with ethanol reduced the association between mTOR and Rheb [77]. Although incubation with leucine alone did not increase the association between these two proteins, it prevented the ethanol-induced decrease in mTOR-Rheb association [77]. The decrease in mTOR-Rheb binding in the presence of ethanol was associated with an ethanol-induced increase in phosphorylation of AMPK^T172^ and TSC2^S1387^, and these ethanol-induced changes were prevented by co-incubation with leucine [77]. The protective effect of leucine on ethanol-induced AMPK phosphorylation occurred in the absence of changes in the concentrations of ATP or AMP. This work suggests that ethanol affects multiple upstream components of mTOR pathway signaling, and leucine appears to protect against these changes without altering the intracellular ATP/AMP ratio. In contrast, other work has observed concomitantly decreased phosphorylation of mTOR^S2448^ and AMPK^T172^ in murine myotubes and SKM from patients with alcohol-related liver disease [120]. Although it is possible that ethanol reduced mTOR-Rheb binding via AMPK/TSC2/Rheb signaling, it is also possible that ethanol suppressed the ability of mTOR to interact with Rheb due, at least in part, to preventing mTOR from binding the Rag complex at the lysosome.

In addition to its effects on the AMPK/TSC2/Rheb and Rag-Ragulator branches upstream of mTOR, evidence exists, albeit mixed, regarding the effects of acute ethanol on components of the PI3K/Akt portion of the pathway upstream of mTOR. In male rats, acute ethanol (75 mmol/kg body mass) did not affect the basal levels or insulin-induced increase in insulin receptor and insulin receptor substrate (IRS)-1 phosphorylation [121]. IGF-1 induced an increase in IGF-1 receptor and IRS-1 phosphorylation, and insulin and IGF-1-induced increases in Akt^T308^ phosphorylation, despite ethanol-induced changes in hormone-stimulated phosphorylation of proteins downstream of mTOR [121]. In contrast, female mice administered an acute dose of ethanol (3 g/kg body mass; blood alcohol concentration (BAC) of approximately 0.27 g/dL) had reduced basal phosphorylation of Akt^T308^ and Akt^S473^ compared to control animals despite greater circulating insulin concentrations 1 h after ethanol administration [122], indicating aberrant insulin signaling that could potentially be due to insulin receptor internalization or a direct effect of ethanol either at or upstream of Akt. The divergent results warrant further investigation regarding the effect of acute ethanol intoxication on PI3K/Akt signaling upstream of mTOR, as the effects could depend on time of muscle excision during the intoxication period, sex, phosphorylation site, and species. Furthermore, male rats administered a dose of ethanol sufficient to achieve a BAC of approximately 0.27 g/dL had lower circulating total IGF-1 and intramuscular free IGF-1 compared to rats administered saline after 2.5 h [112]. Therefore, although limited evidence suggests that ethanol does not affect the IGF-1 receptor responsiveness or Akt phosphorylation after administration of exogenous insulin or IGF-1, it could affect endogenous concentrations of these hormones, potentially leading to reduced hormone-driven signaling upstream of mTOR.

### 3.2. Ethanol Effects Downstream of mTOR

In addition to the upstream alterations in mTOR pathway signaling observed with ethanol, changes downstream of mTOR have also been observed, but not all components downstream of mTOR are similarly and simultaneously affected. Reduced mTOR^S2448^ phosphorylation is consistently detected in C2C12 myocytes incubated with ethanol (100 mM) for 18–24 h [77,115,123], in rats approximately 2.5 h after ethanol administration (75 mmol/kg body mass) [118], and in isolated rat hindlimb muscle perfused with an ethanol-containing buffer (0.25 g/dL) for 2.5 h [111]. In addition to increasing the interaction between mTOR and Raptor, incubation with ethanol also increased the association between Raptor and Deptor (an inhibitory component of both mTOR complexes) in C2C12 myocytes [109], which is consistent with reduced mTOR^S2448^ phosphorylation. Basal phosphorylation of 4E-BP1^T37/46^ is also reduced with ethanol in C2C12 myocytes [77,109,123] and in rodents within 1–8 h after ethanol administration [111,112,118,124]. Similarly, binding of 4E-BP1 with eIF4E increased and binding of eIF4E with eIF4G decreased with ethanol in both model systems [10,112,118,123], indicating potentially reduced capacity for basal translation initiation with acute ethanol. It should be noted that basal 4E-BP1^T37/46^ phosphorylation was not affected by ethanol 1 h after administration in female mice despite a BAC of approximately 0.27 g/dL and an overall reduction in MPS [122], indicating that species (mouse versus rat), timing of muscle collection, and/or sex might play a role in the ethanol-induced changes in signaling.

In contrast to the consistent findings across models for the inhibitory effects of ethanol on mTOR, findings for the effect of ethanol on basal phosphorylation of S6K1, a direct downstream target of mTOR, appear to differ between models. In C2C12 myocytes, reduced S6K1^T389^ phosphorylation in response to incubation with 100 mM ethanol for 18–24 h is consistently observed [77,109,115,123] with concomitant changes in phosphorylation of proteins downstream of S6K1: reduced rpS6^S235/236^ phosphorylation [77,123], reduced eEF2K^S366^ phosphorylation [108], and increased eEF2^T56^ phosphorylation [77,108,123]. On the other hand, in vivo administration of ethanol to rodents did not alter basal S6K1^T389^ phosphorylation after 1–2.5 h [118,121,122,125]. Despite unchanged basal S6K1 phosphorylation, ethanol reduced basal phosphorylation of rpS6^S235/236^ and rpS6^S240/244^ in vivo [112,118,121,122,125]. Interestingly, acute ethanol administration resulted in decreased phosphorylation of eEF2^T56^ in the psoas muscle [126], which is in contrast to in vitro findings, and was associated with enhanced elongation. Therefore, it has been proposed that the in vivo reduction in MPS observed with acute ethanol intoxication is likely due to reduced translation initiation [10] rather than a reduction in efficiency of peptide chain elongation [126]. Although this reasoning is plausible, more research into the acute effects of alcohol on elongation factors in vivo is warranted, as reduced eEF1A content in response to alcohol feeding in rat gastrocnemius has also been reported [127]. Moreover, the reason for the difference in the effects of ethanol on basal S6K1 phosphorylation between model systems is unknown. The difference could be due to alcohol metabolism; however, treatment with 4-methylpyrazole (4-MP), an alcohol dehydrogenase inhibitor, failed to account for the ethanol-mediated decrease in IGF-1-stimulated S6K1 phosphorylation in vivo [125]. Furthermore, the basal decrease in rpS6 phosphorylation with ethanol was still present with 4-MP [125]. Therefore, alcohol metabolism is unlikely to explain basal differences in S6K1 phosphorylation with ethanol between model systems. Since cultured myocytes are actively growing or maturing cells, it is possible that they might simply have higher basal S6K1 phosphorylation compared to mature muscle, and therefore ethanol may have a more measurable impact in the unstimulated state in this model system. The dose- and time-dependent differences in ethanol exposure between cell culture and rodent models may also account for some of the differences observed.

Ethanol can also alter changes in signaling downstream of mTOR in response to the administration of anabolic agents such as leucine, insulin, and IGF-1. For example, co-incubation of C2C12 myocytes with leucine and ethanol in vitro prevented the leucine-induced increases in phosphorylation of mTOR^S2448^, 4E-BP1^T37/46^, S6K1^T389^, and rpS6^S235/236^ [77]. In vivo, ethanol administration prevented the leucine-induced increases in phosphorylation of 4E-BP1^T70^ and γ isoform but not 4E-BP1^T37/46^, S6K1^T389^ and S6K1^T421/S424,^ and rpS6 (site not indicated), and attenuated the leucine-induced increase in phosphorylation of mTOR^S2448^ [118]. Ethanol also reversed the leucine-induced decrease in 4E-BP1 bound to eIF4E, but the amount of eIF4E bound to eIF4G when leucine and ethanol were both administered was not different from either leucine or ethanol alone [118]. However, ethanol prevented the increase in phosphorylation of eIF4G in response to leucine [118], which could interfere with eIF4F complex assembly [128]. Additionally, Lang et al. [125] found that ethanol administration either attenuated (BAC of 0.015 g/dL) or prevented (BAC of 0.165 or 0.290 g/dL) the IGF-1-induced increases in S6K1^T389^ and rpS6^S235/236^ phosphorylation 2.5 h after ethanol administration regardless of route of administration (i.e., oral or intraperitoneal) but did not alter the IGF-1-induced increase in 4E-BP1^T37/46^ phosphorylation or decrease in 4E-BP1 bound to eIF4E. Furthermore, a single dose of ethanol (75 mmol/kg body mass) prevented the IGF-1 induced increases in S6K1^T389^ and rpS6^S235/236^ phosphorylation 1 and 4 h after administration, and these decreases persisted through 8 h after administration [125]. The same dose of ethanol did not affect the IGF-1 induced increase in 4E-BP1^T37/46^ phosphorylation or decrease in 4E-BP1 bound to eIF4E at 1, 4, 8, or 24 h after administration [125]. In a separate but similar investigation, Kumar et al. [121] also found that ethanol (75 mmol/kg body mass) prevented the IGF-1 induced increase and attenuated the insulin-induced increase in S6K1^T389^ phosphorylation. Ethanol also attenuated the insulin-, IGF-1, and refeeding-induced increases in rpS6 phosphorylation [121,124]. However, ethanol did not simultaneously affect increases in 4E-BP1 phosphorylation or eIF4E bound to eIF4G [121,124]. Ethanol administration reversed the decrease in 4E-BP1 bound to eIF4E in response to IGF-1 but not insulin [121], a finding that is different from that of Lang et al. [125] regarding ethanol and IGF-1 despite a much higher dose of IGF-1 in the study by Kumar et al. (25 mmol/kg versus 25 nmol/kg). However, the BAC at the time of sacrifice was slightly higher in Kumar et al. (approximately 0.322 g/dL) compared to Lang et al. (approximately 0.290 or 0.165 g/dL), which might account for at least some of the difference in findings. It should be noted, however, that although Lang et al. [125] observed that the IGF-1-induced reduction in 4E-BP1 bound to eIF4E after an acute dose of ethanol was not different from IGF-1 stimulation alone; it was also not statistically different from rats administered neither ethanol nor IGF-1. Overall, it appears that acute alcohol intoxication has a larger role in suppressing S6K1/rpS6 signaling compared to 4E-BP1/eIF4E signaling in response to hormonal stimulation and suppresses signaling downstream of mTOR in response to leucine stimulation. Conversely, these anabolic agents can decrease or prevent the ethanol-induced changes in signaling downstream of mTOR in preclinical models. These findings provide strong evidence supporting the development of translational studies examining amino acid co-ingestion as a method to ameliorate negative effects of ethanol on SKM mTOR signaling and MPS.

## 4. Resistance Exercise (RE) and Signaling through mTOR

A landmark study by Baar and Esser in 1999 [129] demonstrated, for the first time, that stimulated SKM contractions, designed to mimic RE, induce an acute increase in S6K1 phosphorylation. Moreover, the percent change in phosphorylation of S6K1 after an acute bout of muscle contractions strongly correlated with the percent increase in muscle mass after stimulation 2 times per week for 6 weeks [129]. S6K1 activation and growth were mediated by loading and muscle fiber type, where eccentric contractions (and more force) applied to fast-twitch muscles (extensor digitorum longus and tibialis anterior) had the most robust response, and concentric contractions (and less force) applied to the slow-twitch soleus did not induce growth [129]. This study provided initial evidence for an important role of mTOR pathway signaling in load-induced muscle growth. Administration of the mTOR inhibitor rapamycin to rats in a compensatory overload model blocked overload-induced hypertrophy after 14 days [16,130], and acutely blocked the early (1–2 h and 6 h) increase in MPS in response to a bout of RE in rats and humans [15,131], supporting a key role for mTOR pathway signaling in load-induced muscle hypertrophy. Further work using an inducible raptor knockout model has provided additional support for the necessity of signaling through mTOR within complex 1 in load-induced muscle hypertrophy, although it was not necessary for increased MPS [84]. Therefore, signaling through the mTOR pathway in response to mechanical loading has been a subject of great interest and has been widely studied in rodents and humans. This portion of the review will focus on RE-induced alterations in mTOR pathway signaling in human SKM.

### 4.1. mTOR Signaling Immediately after RE

Immediately after an acute bout of RE, the phosphorylation of upstream components of the mTOR signaling pathway largely indicate inhibitory input toward mTOR in human skeletal muscle. For example, AMPK activity [132] and phosphorylation [133,134,135,136] are increased either immediately following or 10 min after cessation of RE in non-RE-trained individuals, whereas it appears that RE training blunts the RE-induced increase in AMPK phosphorylation [133]. In untrained individuals, the RE-induced AMPK^T172^ phosphorylation can persist at 1 h after exercise in the fasted state [15,132]. This is likely due to high ATP demand during RE and thus a reduced ATP:AMP ratio [137], which is a major driver of AMPK phosphorylation and activation [138]. Furthermore, glycogen synthase kinase (GSK)-3β^S9^ phosphorylation, which inhibits mTOR signaling by phosphorylating and activating TSC2 [139], was increased immediately after RE in the fed state [136]. Decreased phosphorylation of Akt^T308^ and Akt^S473^ has also been observed immediately after RE [140]; however, this finding is inconsistent as others have reported no change in Akt [132,135] or TSC2^T1462^ (an Akt target) [132] phosphorylation immediately to 10 min after RE compared to rest. Regardless, despite increased AMPK phosphorylation and potentially decreased or unchanged Akt phosphorylation immediately after RE, mTOR^S2448^ and S6K1^T389^ phosphorylation appear unchanged immediately to 10 min after RE in the fasted state [132,135,140]. However, increases in mTOR and S6K1 phosphorylation have been reported as early as 15 min after RE in the fasted state [141,142], indicating a temporal shift toward anabolic signaling, and immediately after RE in the fed state [136]. Decreased 4E-BP1^T37/46^ phosphorylation immediately to 10 min after RE has been reported [132,135,140], which is consistent with increased AMPK phosphorylation at that time point and suggests that the formation of eIF4F, and thus increased translation initiation to allow for the increased rate of MPS observed with RE, have a delayed response after RE. Consistent with these observations, the rate of MPS throughout to immediately after RE is decreased compared to baseline [132]. Importantly, when recreationally trained participants performed RE, TSC2/Rheb colocalization was decreased and Rheb/mTOR colocalization was increased 10 min after exercise compared to rest [49], suggesting that RE stimulates intracellular conditions that promote mTOR activation immediately after exercise even if that increased signaling is not yet observed. Overall, it appears that mTOR signaling is largely inhibited immediately after RE when performed in the fasted state, and especially in untrained individuals, but feeding and training status can potentially alter these responses. Further investigation of the early signaling responses in fed and fasted states, in trained and untrained individuals, and the influence of immediate post-exercise mTOR pathway signaling on MPS would be valuable additions to the literature.

### 4.2. mTOR Signaling in Recovery from RE and Influencing Factors

In contrast to immediately after exercise, mTOR pathway activation is upregulated later into the RE recovery period (e.g., ~1–6 h after RE). For example, in untrained, fasted individuals, mTOR^S2448^ [15,132,141,143,144,145], S6K1^T389^ [15,132,135,141,144,145], and rpS6^S235/236^ and rpS6^S240/244^ [15,141,143] phosphorylation are increased and eEF2^T56^ [15,132,141,144] phosphorylation is decreased 1–4 h after exercise. These results correspond with increased MPS within the first 2 h after exercise [15,132,144]. Thus, despite the lack of a feeding stimulus, RE alone stimulates mTOR pathway signaling and MPS in the post-RE recovery period, at least in untrained human participants.

#### 4.2.1. Influence of Feeding

Although conducting investigations in which participants remain fasted allows for elucidation of the specific effects of RE on intramuscular signaling and MPS without the influence amino acid- or insulin-induced signaling, this design is likely less externally valid compared to a design which incorporates feeding before and/or after the exercise bout. Furthermore, RE and feeding each stimulate mTOR pathway signaling and MPS, and the combined effects are greater than either stimulus alone [143,146,147,148]. For example, Moore et al. [147] observed increased S6K1 phosphorylation 1 h after feeding alone or RE followed by feeding in human participants. Only when RE was performed prior to feeding, increases in S6K1 phosphorylation were still present at 3 and 5 h. At all post-feeding time points (1, 3, and 5 h), eEF2 phosphorylation was reduced for feeding combined with RE compared to feeding only, but 4E-BP1 phosphorylation was similarly elevated in both groups [147]. Altogether, these results indicate a stronger and prolonged anabolic stimulus with RE and feeding combined compared to feeding alone. Furthermore, ingestion of 15 g of whey protein after fatiguing lower body RE increased the rate of MPS (myofibrillar fraction) compared with whey ingestion alone [149]. Song et al. [49] observed increased activation of S6K1 at 1 and 3 h after human participants performed RE with or without feeding, but the magnitude of the elevation was much greater at 1 h when feeding followed RE. Although mTOR colocalization with eIF3F increased in both groups at 10 min, 1 h, and 3 h after RE, the magnitude of the increase was greater in the fed group 1 h after RE [49]. A supplement containing 10 g of whey protein and 10 g of leucine ingested after RE augmented phosphorylation of rpS6 to a greater extent than placebo (no nutrients) 45 min after exercise, and induced Akt phosphorylation, likely due to insulin signaling, that was absent in the placebo condition [150]. With amino acid administration either immediately [151] or 1 h [146] after RE, the increases in mTOR activation [151] and rate of MPS [146] were greater than with placebo. Moreover, when 20 or 40 g of egg protein was ingested after RE, the rate of MPS from 1–4 h after RE was greater than when only up to 10 g of egg protein was ingested after RE [152], suggesting a dose-response relationship between ingested protein and augmented rates of MPS where sufficient protein is required to confer maximal benefits. Overall, although RE and feeding each independently stimulate mTOR pathway signaling and MPS, the anabolic effects are greater when combined.

#### 4.2.2. Influence of Age

Age also affects anabolic responses to an acute bout of RE in the fed [153] and fasted [154] states. When younger (approximately 30 years) and older (approximately 70 years) men with similar body mass ingested the same dose of 20 g of essential amino acids after RE, similar increases in mTOR^S2448^ and S6K1^T389^ phosphorylation at 1, 3, and 6 h and similar decreases in eEF2^T56^ phosphorylation at 3 and 6 h into recovery were observed, but the increased 4E-BP1^T37/46^ phosphorylation observed in both groups at 3 h persisted to 6 h after RE only for the younger men [153]. Since 4E-BP1 inhibits translation initiation, the rate-limiting step in protein synthesis, the difference in 4E-BP1 phosphorylation between younger and older men could be important for subsequent rates of MPS. Indeed, MPS was increased in the younger group from 1–3 and 3–6 h after RE, but an increase in MPS in the older group was not observed until 3–6 h after RE [153]. It would be beneficial to determine what differences in MPS between younger and older individuals, if any, exist beyond 6 h after RE and feeding as increases MPS can persist well beyond that time point following RE [155]. Attenuation of the dose-dependent amino acid-induced increases in mTOR pathway signaling and MPS (myofibrillar and sarcoplasmic fractions) has been observed in older versus younger men in the absence of RE and with an insulin clamp [156] suggesting reduced amino acid sensitivity in the SKM of older individuals at rest, but timing of protein intake after RE could, at least in part, alleviate this reduced sensitivity to amino acids. When older men ingested protein immediately after each RE session during 12 weeks of RE training, muscle size increased as a result of training, whereas no increase in muscle size was observed when the same protein bolus was ingested 2 h after each RE session [157]. Dose-dependent increases in MPS were observed when older men ingested up to 45 g of protein after exercise [158]. For older women, a lower protein dose (10 g) matched for leucine (3 g) was as effective as a higher protein dose (25 g) at increasing MPS after RE, although the higher protein dose was required for increasing MPS without exercise [159]. Sex differences in anabolic resistance among older individuals have yet to be examined, and studies among older women are lacking. Together, existing evidence suggests that mTOR pathway signaling and MPS in response to RE and amino acids are reduced or delayed in older men compared to younger men. Ingesting a sufficient dose of protein and timing this dose immediately after cessation of RE could aid in improving anabolic signaling and MPS in older individuals. For an in-depth review on leucine-mediated MPS in the context of aging, see reference [160].

#### 4.2.3. Influence of RE Programming

Acute program variables such as volume and intensity of exercise are also important factors in the magnitude of the anabolic response to RE. Performance of 3 sets versus 1 set of lower body resistance exercise elicits a more substantial increase in myofibrillar MPS, increases rpS6^S240/244^ phosphorylation 5 h after exercise, and increases the duration of enhanced sensitivity to amino acids (e.g., the following day) [161]. Similarly, 4 sets of unilateral leg extensions performed to failure at 90% and 30% of 1-repetition maximum (1RM) elicited significant increases in myofibrillar MPS and Akt^S473^ and mTOR^S2448^ phosphorylation, respectively, one day following RE, whereas performance of a work-matched leg extension protocol using 30% of 1 RM (i.e., not performed to momentary muscle failure) did not result in such increases [149]. Therefore, the load might be of less importance than achieving momentary muscular fatigue with sufficient volume. However, only 3–4 sets of lower body exercise is still lower volume than a typical RE session for a trained individual. Performing 5 lower body exercises for 4–6 sets each using a high volume (10–12 repetitions per set at 70% of 1RM) or a high intensity (3–5 repetitions per set at 90% of 1RM) protocol induced increases in phosphorylation of rpS6 at 1 and 5 h after exercise, but only the high volume protocol induced increased IGF-1 receptor phosphorylation at 1 h after RE [162]. Interestingly, both protocols resulted in decreased mTOR^S2448^ and Akt^S473^ phosphorylation 5 h after RE, possibly due to the combination of strenuous RE and low nutrient intake provided to participants [162]. Therefore, the signaling responses to fatiguing high volume and high intensity RE did not substantially differ. A comparison of such protocols with sufficient nutritional intake (or at least protein provision) after RE, especially with a measure of MPS and mTOR pathway signaling, would provide valuable insight into the signaling associated with high volume versus high intensity RE with greater external validity.

#### 4.2.4. Influence of Training Status

Training status also influences mTOR pathway signaling and MPS in response to RE. Only a few investigations have directly compared acute responses to RE in trained and untrained SKM, but several of these studies have demonstrated a blunted anabolic response in trained muscle. For example, when rats performed an acute bout of stimulated muscle contractions (designed to mimic RE) in an untrained state, S6K1^T389^ and rpS6^S235/236^ phosphorylation increased to a greater extent than in those who had undergone 12 or 18 bouts of stimulated muscle contractions (to mimic RE training; 1 bout performed every other day with stimulation voltage and frequency adjusted to achieve maximal isometric force) [163], indicating an attenuated signaling response in trained SKM. In humans, fed-state RE resulted in increased S6K1^T389^ phosphorylation immediately and 4 h after exercise in untrained SKM, but only immediately after exercise in trained SKM using the same relative workload [136]. Similarly, rpS6^S235/236^ phosphorylation was increased 4 h after exercise in untrained muscle, but this increase was not observed after training regardless of whether a constant or progressive loading scheme was used [136]. However, when RE training was followed by a 10-day detraining period, RE once again induced S6K1^T421/S424^ and rpS6^S235/236^ phosphorylation similar to that of the first RE session [164]. Strength re-testing to determine loads for the post-detraining measurement was not reported, but participants reportedly performed 3 sets of 8–12 repetitions at pre-training 10-repetition maximum with load adjustments as needed for the progressive loading group [164].

The protein synthetic response also appears to become more targeted after RE training. For example, when untrained individuals performed an acute bout of RE, MPS increased for the myofibrillar and the mitochondrial fractions, but after training, only an increase in myofibrillar MPS was observed [136]. Furthermore, when young men trained only 1 leg and performed a bout of unilateral leg extensions using each leg, the increase in mixed muscle MPS was greater in the trained leg than the untrained leg 1–4 h after RE, but by 24–26 h after RE, MPS was elevated above baseline only in the untrained leg even with the trained leg load adjusted for most recent working load [165]. Others have also found a greater elevation of mixed muscle MPS in untrained SKM after acute RE [166], whereas the increase in myofibrillar MPS does not appear to differ between the trained and untrained states [166,167] at the same relative workloads. Therefore, there may be a difference in response to training between muscle protein fractions. It is possible that the prolonged increase in MPS in the untrained leg resulted in greater overall protein synthesis for that leg. However, it should be noted that untrained muscle subjected to a bout of RE also increases MPB measured by primed constant infusion of 15N-phenylalanine to a greater extent than trained muscle at the same relative workload [168]. For this reason and others not mentioned herein, it has been hypothesized that the initial greater anabolic signaling and MPS observed in untrained individuals is targeted at repair of damaged tissue, whereas the more subtle increase in anabolic signaling and MPS observed after RE training might be more specific and focused on true hypertrophic adaptations (reviewed in detail in reference [169]). It should also be noted that basal MPS is increased following RE training [166,170].

Overall, acute RE appears to result in decreased mTOR pathway signaling during and immediately after exercise with a concomitant decrease in MPS followed by increased mTOR pathway signaling during the 6 h after exercise. Moreover, given sufficient volume, an acute bout of RE appears to increase the anabolic responsiveness of muscle to amino acids at least through 29 h after exercise. Factors such as nutrition, age, acute program variables, and training status impact the magnitude of mTOR pathway signaling responses, rates of MPS, and the duration of the elevation in MPS.

## 5. Combination of Ethanol and RE on Signaling through mTOR

**Figure 2:**
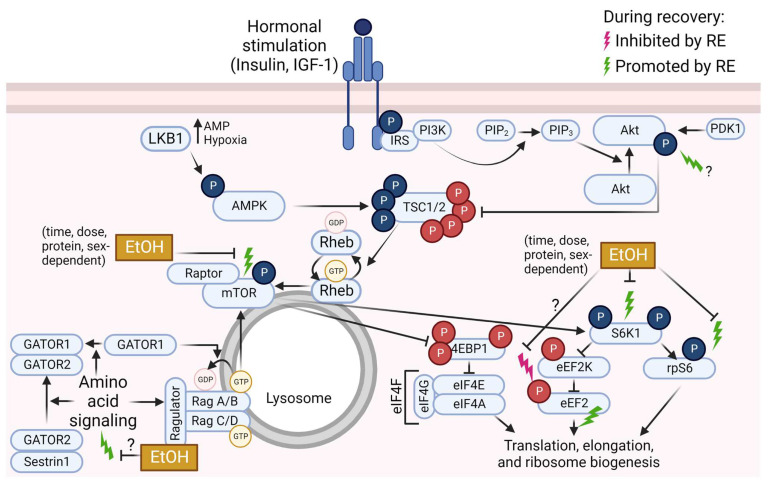
Overview of mammalian/mechanistic target of rapamycin (mTOR) signaling pathway events that are inhibited (pink lightning bolt) or promoted (green lightning bolts) by resistance exercise (RE), and known or potential impacts of ethanol (EtOH) on these changes. Phosphate groups (P) in red indicate inhibitory events; P in blue indicate activating events. Abbreviations: 4E binding protein 1 (4EBP1), AMP-activated protein kinase (AMPK), diacylglycerol kinase (DGK), diacylglycerol (DAG), eukaryotic elongation factor (eEF), eukaryotic initiation factor (eIF), GAP activity toward Rags (GATOR), insulin receptor substrate (IRS), insulin-like growth factor-1 (IGF-1), liver kinase B1 (LKB1), p70 S6 kinase 1 (S6K1), phosphatidic acid (PA), phosphatidylcholine (PC), phosphatidylinositol (4,5)-bisphosphate (PIP2), phosphatidylinositol (3,4,5)-trisphosphate (PIP3), phosphoinositide 3-kinase (PI3K), phospholipase D (PLD), protein kinase B (Akt), pyruvate dehydrogenase kinase 1 (PDK1), Ras homolog enriched in brain (Rheb), ribosomal protein S6 (rpS6), tuberous sclerosis complex (TSC). Created using Biorender.com (accessed on 13 December 2022).

As described above, acute ethanol and RE independently impact mTOR pathway signaling, but in opposite directions such that acute ethanol generally has suppressive effects, whereas RE generally has stimulatory effects. In contrast to the independent effects of ethanol and RE on mTOR pathway signaling, few studies have investigated the combined effects of ethanol and RE on mTOR pathway signaling and MPS. Recreational ethanol use is higher among physically active individuals compared to sedentary individuals [6,7,8], especially for those who participate in RE and vigorous-intensity exercise [8,9]. The opposing independent effects of ethanol and RE on mTOR pathway signaling and MPS suggest that alcohol may interfere with exercise adaptations in athletes and active individuals. Moreover, muscle hypertrophy is an adaptation to RE training, making this training style an attractive, low-risk option for counteracting myopathy associated with at-risk alcohol use. For example, we previously found that 6 weeks of RE training increases muscle size and strength in men undergoing treatment for substance misuse, many of whom cited alcohol as a primary or secondary drug of abuse [171]. However, due to the general inhibitory effects of acute alcohol on mTOR pathway signaling, the efficacy of RE to improve muscle mass and function in people with alcohol-associated myopathy may differ based on whether alcohol is consumed in the peri-RE period. For these reasons, the combined effects of alcohol (ethanol) and RE on mTOR pathway signaling and MPS have recently drawn attention from the scientific community. For a summary of mTOR pathway signaling during the post-RE period and influences of ethanol, see Figure 2.

### 5.1. Findings from Preclinical Studies

The combined effects of ethanol and RE on mTOR signaling have been investigated in rodents and in humans. In the first rodent study examining this question, ethanol (3 g/kg) was administered prior to unilateral electrically stimulated muscle contractions and resulted in attenuation or prevention of contraction-induced S6K1^T389^, 4E-BP1 (γ isoform), and rpS6^S240/244^ phosphorylation 30 min after muscle contraction [19]. By 4 h after contractions, contraction-induced increases in S6K1^T389^, rpS6^S240/244^, 4E-BP1 (γ isoform), and mTOR^S2448^ were absent in the rodents who received alcohol prior to contraction [19]. By 12 h after contractions, the contraction-induced increases in S6K1^T389^ and rpS6^S240/244^ were still prevented or attenuated, respectively, in rats who had been administered ethanol [19]. Similar to previous work, ethanol suppressed basal rates of MPS compared to controls, and also prevented contraction-induced changes in MPS observed at 30 min and 4 h after muscle contractions in control rats [19]. By 12 h after contractions, MPS was greater in the stimulated leg compared to the non-stimulated leg in both groups, but the magnitude of MPS was less in both legs in the ethanol-treated rats compared to control rats [19]. Thus, ethanol administered before muscle contraction in fasted rats that remained fasted throughout recovery from exercise reduced contraction-induced mTOR signaling and MPS for up to 12 h after exercise. In contrast, the same relative amount of ethanol administered to mice 2 h after electrically-stimulated muscle contraction did not prevent the contraction-induced increase in S6K1^T389^, rpS6^S235/236^ and rpS6^S240/244^, or 4E-BP1^T37/46^ phosphorylation 4 h after contractions [20]. However, ethanol administration prevented the contraction-induced reduction in eEF2^T56^ phosphorylation [20]. Despite minimal disturbance to mTOR pathway signaling at that time point, basal MPS was suppressed and the contraction-induced increase in MPS at 4 h was prevented in ethanol-administered mice [20], possibly due, at least in part, to suppressed rates of peptide chain elongation evidenced by reduced activation of eEF2. In rodents, binge-like ethanol administration before or after electrically stimulated muscle contractions suppresses contraction-induced increases in MPS for at least 4 h, but the effects on mTOR pathway signaling appear to differ based on timing of ethanol administration and possibly species.

### 5.2. Findings from Clinical Studies

Several investigations of the effects of ethanol on exercise-stimulated mTOR pathway signaling and protein synthesis have been carried out in humans. For example, ethanol (1.5 g/kg over 3 h, peak BAC ~0.06 g/dL) co-ingested with protein after a bout of concurrent RE and aerobic exercise attenuated the exercise-induced increase in mTOR^S2448^ phosphorylation 2 h, but not 8 h, after exercise and the rate of myofibrillar MPS measured between 2 and 8 h after exercise compared to post-exercise protein ingestion, but did not affect S6K1^T389^ or 4E-BP1^T37/46^ phosphorylation [18]. Interestingly, eEF2^T56^ phosphorylation was reduced at 2 and at 8 h after exercise only when ethanol was consumed post-exercise [18], which is in contrast to expectations given the reduced rate of MPS observed with ethanol and preclinical findings. The overall results of this study suggest a suppressive effect at least on the MPS and a minimal effect on mTOR pathway signaling in the context of combined RE and aerobic exercise when protein is consumed post-exercise. However, aerobic exercise and RE can (but do not always) elicit divergent intramuscular signaling [172] and fractional MPS [136] responses that correspond with specific training adaptations. In an attempt to better elucidate the specific effects of ethanol on RE-induced mTOR signaling, a high-volume, fatiguing resistance exercise protocol was used [17]. Consuming ethanol (1.09 g/kg fat-free body mass over 10 min, peak BAC ~0.11 g/dL) after RE attenuated the RE-induced increase in mTOR^S2448^ and S6K1^T389^ phosphorylation 3 h after exercise in men but not in women [17]. These ethanol-induced effects were no longer significantly different by 5 h post-exercise. In a second study utilizing the same exercise protocol, men co-ingested whey protein with ethanol after exercise. RE with protein increased mTOR^S2448^, S6K1^T389^, rpS6^S235/236^, and eEF2K^S366^ phosphorylation and decreased eEF2^T56^ phosphorylation 2 h after RE; however, ethanol did not affect these results (unpublished observations). One major difference between the two studies was nutrient timing. In the first, a standardized meal was administered 65 min prior to exercise (4 h before the first post-exercise biopsy) [17], so it is possible that the augmented signaling due to amino acids was no longer observed by the time of sample collection. In the second, a bolus of whey protein was administered immediately after RE (2 h before the first post-exercise biopsy; unpublished observations). Whey protein substantially augments RE-induced mTOR signaling responses and MPS [146,150,173]. Therefore, it is possible that the combined anabolic effect of strenuous RE and whey protein supplementation was greater than the potential anti-anabolic effect of ethanol at the dose administered and the time of muscle sample collection. Given the divergent results of the studies regarding the combined effects of ethanol and RE in humans, future research should investigate the effects of acute program variables, ethanol dose, and peri-exercise nutrient timing in the context of ethanol and RE.

### 5.3. Implications for Chronic Ethanol on RE Training-Induced Adaptations

There is substantial evidence regarding independent and opposite acute effects of ethanol and RE on mTOR pathway signaling, and some evidence supporting acute ethanol attenuating the RE-induced increase in activation of this pathway. Therefore, there is reason to believe that regularly consuming ethanol during the post-RE period could negatively impact hypertrophic adaptations. To date, no study has evaluated this question in humans using traditional RE training, and few studies have examined this or similar questions in preclinical models. The single study directly examining whether chronic ethanol impacts overload-induced muscle growth found that ethanol consumption described as moderate (~20 g/kg/day for rodents) did not attenuate overload-induced hypertrophy (i.e., synergist ablation) in male mice [174]. Others have examined the impacts of chronic ethanol on muscle mass recovery after immobilization-induced atrophy. In female rats, recovery of muscle mass was not observed 14 days post-immobilization regardless ethanol intake [175]. However, in male rats, twice daily ethanol (BAC peak ~0.15 g/dL) impaired recovery of muscle mass 5 days after immobilization-induced atrophy in association with decreased mTOR pathway signaling in rats [176]. Whether heavier drinking patterns impact overload-induced muscle growth in males or females remains to be studied.

Only the BEER-HIIT study has evaluated the impacts of regular ethanol consumption on exercise induced adaptations in humans [177,178], and the exercise intervention was high-intensity interval training (HIIT) rather than traditional RE training. In that study, relatively moderate ethanol intake (1.7–2.6 and 0.9–1.7 standard drinks per day for men and women, respectively) did not negatively impact increased lean mass [177] or hand grip strength [178] in response to 10 weeks of HIIT. It should be noted that these results cannot be generalized to ethanol intake that exceeds moderate levels (i.e., heavy or binge drinking) or to traditional RE training interventions. Together with the preclinical studies, the BEER-HIIT study results provide promising evidence that exercise interventions could be effective for combating alcohol-induced muscle loss even in active drinkers. However, the ethanol intake threshold that would negatively affect growth responses is unknown. The impact of chronic ethanol consumption on long-term training adaptations in healthy individuals (e.g., not yet experiencing atrophy or myopathy) or athletes is unknown. Due to ethical considerations, examining the effects of heavier drinking patterns on mTOR-mediated RE training-induced adaptations would need to be performed either (1) with participants who already drink more than moderately or (2) using preclinical models. Both approaches would add value to the literature.

## 6. Conclusions

Alterations in SKM size are determined by the relative rates of MPS and MPB over time. The mTOR signaling pathway has been identified as an important mediator of the translational process, is critical for SKM hypertrophy, and is controlled by hormones, nutrients, and mechanical stimuli. However, the specific mechanisms by which amino acids and mechanical stimuli activate the mTOR pathway remain to be fully elucidated. Acute ethanol ingestion suppresses MPS and components of the mTOR signaling pathway, and the exact mechanism for this suppression is also not yet established. Notably missing from the literature are dose-response studies examining the impact of different amounts of ethanol on mTOR pathway signaling. Whether differences exist based on the type of ethanol-containing beverage consumed (e.g., liquor, beer, wine) should also be explored since other beverage components may have confounding impacts on mTOR signaling and MPS. In contrast to ethanol, acute RE increases the rates of MPS and MPB, but given sufficient nutrients, net protein balance is positive after RE. Acute RE also increases mTOR pathway signaling. Few investigations have explored the combined effects of ethanol and RE on mTOR pathway signaling and MPS. Overall, it appears that ethanol can, but does not always, suppress RE-induced increases in mTOR pathway signaling and protein synthesis, and this suppression is likely dependent on the specific exercise protocol, timing of ethanol intake, dose of ethanol, nutrient co-ingestion, and sex of the individuals. Since increased mTOR pathway signaling can persist long after a bout of RE, and ethanol may not be consumed in the early post-RE period, the impact of ethanol consumed later in the recovery period (e.g., morning RE and evening ethanol intake) on mTOR pathway signaling in skeletal muscle would be highly relevant. The effects of ethanol on RE-induced anabolic signaling should be further explored since RE is a potent stimulus for increasing muscle size and could be a useful method of counteracting and preventing muscle wasting in at-risk populations such as those with alcohol use disorder.

## Data Availability

Not applicable.
